# Training-induced Changes in Development and Relaxation Electromechanical Delay (EMD)

**Published:** 2018-03

**Authors:** Min-Ho SONG, Tae-Young KIM, Sang-Ho LEE

**Affiliations:** 1. Dept. of Liberal Arts, Korean Langage and Literature, Hongik University, Seoul, Korea; 2. College of Education, Hankuk University of Foreign Studies, Seoul, Korea

## Dear Editor-in-Chief

Electromechanical delay (EMD) means a lag time between the onset of electrical activity and force output (1). In case of the EMD phenomenon, the time when force starts to be produced is called “development EMD (d-EMD)”, while the time when force ends is called “relaxation EMD (r-EMD). This phenomenon indicates that electrical activation occurs before force is produced in case of muscle contraction. However, the EMD phenomenon can occur in many ways depending on gender, age, exercise type and muscle location and it is closed related to the neuromuscular system. Moreover, EMD values can vary depending on the type of muscle contraction (3), and they can vary even in the same examine depending on muscle location (4).

As mentioned above, various studies have been conducted on EMD values, and although many studies have been carried out on d-EMD, which occurs in the early stage of muscle contraction, whereas there have not been enough studies on r-EMD, which occurs at the end of muscle contraction. Especially there is a serious lack of studies on changes in r-EMD depending on the period and intensity of resistance training.

Against this background, this study examined d-EMD and r-EMD occurring during maximal isometric flexion of the biceps brachii in order to offer basis data for application to sports field sites and patients at a clinical stage. Those subjects who participated in the study were male university students who had not participated in regular physical activities for more than the last one month, and who had an average age (22.8 yr), height (175.9 cm), and body weight (67.2 kg).

In terms of experiment method for this EMD study, the bipolar method was used by attaching electrodes to the muscle belly of the subjects, who sat on the complex muscle strength system and started performing maximal isometric flexion with the upper arms maintained at 90 degree angle. At this moment, we measured the electromyograph on the biceps brachii and the muscle strength exerted by the writs before training and in 4 and 8 weeks of training. The muscle resistance exercise program was carried out three times per week for the entire period of 8 weeks by having the subjects perform isometric training at 70% intensity of 1RM (repetition maximum) while sitting on the fixed complex muscle strength system. We measured d-EMD and r-EMD before training and in 4 and 8 weeks of training, and the results show that the d-EMD values decreased by a significant level after 8 weeks compared to those before training (*P*<.001), whereas the r-EMD values showed a significant decrease after 4 and 8 weeks of training compared to those before training (*P*<.001) (Table 1). The EMDs are indicators related to the stiffness of muscle and tendon.

**Table 1:** The values of d-EMD and r-EMD through training

	***Pre-training***	***Post 4 wk***	***Post 8 wk***
d-EMD(m/sec)	22.3 ± 3.7	20.9 ± 4.1	18.3 ± 5.0^***^
r-EMD(m/sec)	29.0 ± 4.3	26.7 ± 3.2^*^	24.8 ± 4.4^***^

* *P*<.05,

*** *P*<.001,

d-EMD: development EMD, r-EMD: relaxation EMD

d-EMD and r-EMD values before and after static stretching might be related to the muscle-tendon unit (MTU), indicating that the force from muscle to bone is increased by training, it can reduce EMD values. As muscle stiffness is dependent on resistance exercise load, a high intensity of exercise can improve the adjustability of nerve and muscle, which can in turn influence the neuro-muscular system.

During maximal voluntary isometric contraction of lower extremity muscle, EMD values were observed to get reduced with an increase in exercise loads in vastus lateralis and rectus femoris. And r-EMD values showed differences before and after fatigue induced by exercise (5), and this is because the reduction in muscle contraction strength can have an influence on the neuromuscular system. Therefore, it means that the EMD values enables a simple and quick evaluation of muscle stiffness, and it can be used as an index of the functional status of the neuromuscular system (6), it can be utilized an indicator to evaluate muscle contraction ability at sports fields and also as an indicator for treatment of neuromuscular disease patients.

In future, more studies need to be followed to identify an optimal exercise load contributing to a significant decrease in EMD values during isometric contractions.
